# Comparative analysis of clinical feature–based machine learning models for predicting myofascial pelvic pain syndrome: a single-center retrospective study

**DOI:** 10.3389/fmed.2025.1689480

**Published:** 2025-12-17

**Authors:** Zhuyin Li, Wenjing Li, Jie Huang, Shuangyu Zhang, Ruolin Jia, Dongxia Liu, Yanhua Dong, Hongguo Zhao, Manman Nai, Lei Li, Hang Yu

**Affiliations:** 1 Department of Obstetrics and Gynecology, The Third Affiliated Hospital of Zhengzhou University, Zhengzhou, China; 2 Prenatal Diagnosis Center, Reproductive Medicine Center, The First Hospital of Jilin University, Changchun, China

**Keywords:** myofascial pelvic pain syndrome (MPPS), modified Oxford muscle strength classification, pelvic floor pressure assessment, predictive modeling, machine learning

## Abstract

**Background:**

Myofascial pelvic pain syndrome (MPPS) is a common but often underdiagnosed cause of chronic pelvic pain in women, significantly affecting quality of life. Early and accurate identification of patients at risk is essential for improving treatment outcomes and reducing the clinical burden.

**Objective:**

This study aimed to develop an effective machine learning-based prediction model for MPPS among Chinese women to assist in early diagnosis and personalized treatment.

**Methods:**

A total of 1,136 women diagnosed with MPPS and 1,448 healthy women who underwent pelvic floor screening during the same period were included, yielding 2,584 samples. Six machine learning algorithms—logistic regression, support vector machine (SVM), random forest (RF), extreme gradient boosting (XGBoost), light gradient boosting machine (LightGBM), and adaptive boosting (AdaBoost)—were trained using 5-fold cross-validation and grid search. Model performance was evaluated using the confusion matrix, precision, recall, F1 score, overall accuracy, and receiver operating characteristic (ROC) curves.

**Results:**

The accuracies of the six models were 0.77, 0.80, 0.91, 0.89, 0.88, and 0.81, respectively. The average area under the ROC curves (AUCs) were 0.670, 0.672, 0.956, 0.951, 0.952, and 0.836, respectively. Among the models, RF achieved the best performance for predicting MPPS, while XGBoost and LightGBM performed slightly lower, with all three models having AUCs above 0.95.

**Conclusion:**

Machine learning models, particularly the random forest algorithm, demonstrated strong potential for accurately predicting MPPS, supporting early diagnosis and enabling personalized clinical decision-making for women affected by this condition.

## Introduction

Myofascial pelvic pain syndrome (MPPS) is a recurrent chronic pain syndrome of the pelvic floor myofascial or related soft tissues, with the main clinical manifestations of pain, muscle stiffness, and spasm in the fascia of the lower back, sacrum, buttocks, and legs, often accompanied by highly sensitive myofascial trigger points, which is harmful to women's physical and mental health (1). The etiology of MPPS is still unclear, and it can exist independently or co-exist with vaginal spasms, dysmenorrhea, endometriosis and other disorders. The symptoms have no distinctive features, which makes it difficult to confirm the diagnosis and easy to misdiagnose, and drugs, psychological interventions, and physical treatments are mostly used in clinics at present (2). In recent years, with the progress of social economy and the pursuit of quality of life, pelvic floor health has received more attentions. However, due to the lack of specific laboratory or imaging manifestations, the lack of uniform diagnostic criteria for MPPS is prone to misdiagnosis and underdiagnosis (3), resulting in a convoluted treatment process, prolonged treatment time, and uncertain treatment outcomes for MPPS patients. Recent studies have highlighted the complex interactions between core muscle dysfunction, pelvic floor health, and postpartum musculoskeletal changes. For instance, a recent randomized controlled trial comparing health parameters in postpartum women with diastasis recti demonstrated that surface electromyography biofeedback-assisted core strengthening exercises improved pelvic floor function and related health outcomes (4). These findings suggest that neuromuscular rehabilitation approaches targeting core and pelvic floor coordination may also be relevant to understanding and managing MPPS. Early diagnosis can help doctors develop more targeted treatment plans, reduce patients' time costs, and improve the quality of life of women with MPPS.

Some medical fields are already exploring the use of AI technology to improve clinical decision-making, such as assisting in disease diagnosis, implementing precision medicine (5) and drug development (6). Since AI can easily process big data to mine the deep information in the data, it has achieved remarkable results in improving medical efficiency and accuracy. In the previously reported literature, a research team has incorporated various clinical characteristics, pelvic floor pressure assessment, and muscle grading assessment data to predict the risk of MPPS development and provide a theoretical basis for the clinical diagnosis of MPPS (7), but the model established is relatively homogeneous. In this study, we use logistic regression (logistic), Support Vector Machine (SVM), Random Forest (RF), Extreme Gradient Boosting (XGBoost), Light Gradient Boosting Machine (LightGBM), and Adaptive Boosting (AdaBoost) machine learning algorithms, aiming to construct a machine learning model for predicting the risk of disease occurrence before the start of MPPS treatment from rich clinical features, comparing the performance of different algorithms on the problem. And select the model with the best comprehensive performance and analyze the importance of different feature variables in the model, in order to assist doctors in identifying MPPS based on the early clinical manifestations of patients, provide personalized treatment for patients, alleviate patients' pain and improve their quality of life.

## Subjects and methods

### Subjects of the study

This study was approved by the Ethics Committee of the Third Affiliated Hospital of Zhengzhou University (Ethics Approval No. 2023-206-01, Approval Date: 2023-08-15), and all methods were performed in accordance with relevant guidelines and regulations. This was a retrospective study that included 2,584 female MPPS patients who were treated at the Third Affiliated Hospital of Zhengzhou University from January 2023 to January 2025 and underwent routine examinations. Among them, 1,136 MPPS patients and 1,448 non-MPPS patients.

### Inclusion and exclusion criteria

Inclusion criteria: (1) diagnosis of MPPS is primarily clinical and based on the following criteria: chronic pelvic pain: persistent or recurrent pain localized to the pelvic region for ≥6 months. Myofascial trigger points (MTrPs): presence of one or more palpable taut bands or nodules within the pelvic floor muscles (e.g., levator ani, obturator internus, coccygeus, piriformis). Reproduction of characteristic pain: digital palpation of the trigger point reproduces the patient's typical pain or referred pain pattern. Local twitch response or tenderness: palpation may elicit a local twitch or spasm in the involved muscle. The diagnosis was confirmed independently by two experienced obstetrician-gynecologists specializing in pelvic pain, based on the above criteria (8); (2) not having received any other treatment related to this disease in the month prior to their visit to our hospital; (3) age ≥18 years old; and (4) having a history of sexual intercourse and being able to undergo vaginal examination.

Exclusion criteria: (1) patients with chronic pelvic pain (CPP) due to known factors such as infection, adenomyosis, fibroids, etc.; (2) patients with acute infectious diseases or other organic diseases; (3) patients during pregnancy or within 3 months postpartum; (4) patients with implanted cardiac pacemakers, metal intrauterine devices, or allergy to electrical stimulation; (5) patients with active vaginal bleeding, local pelvic skin/mucosal injury or infection; (6) patients with other pelvic floor dysfunctional disorders such as urinary incontinence or pelvic organ prolapse; (7) patients with neurologic or psychiatric disorders or other serious illnesses that may prevent cooperation; (8) patients with a history of intervertebral disc disease, sciatica, or other neurologic disorders; and (9) patients with incomplete clinical baseline data.

### Data collection

The target variable of this study is the presence of MPPS. Clinical data of all study subjects were included in this study. Characteristic variables are screened from all variables in order to screen out the characteristics that do not contribute much to the model, and retain the important variables with predictive value to improve the predictive performance of the model. Among them, this study collects case data of study participants, including age, height, weight, body mass index (BMI), pregnancy history and delivery history.

Participants included in the study were assessed for pelvic floor pressure by a trained pelvic floor care therapist, and the assessment was performed using a Myotrac biofeedback device manufactured by Nanjing Weiss Medical Technology Co Ltd (7), which automatically recorded the mean value and coefficient of variation of the pre-rest phase, the maximal value and the time of relaxation. The rapid contraction phase, the mean value, coefficient of variation and the time of relaxation. The endurance contraction phase and the resting mean values and coefficients of variation for the post-contraction phase.

The modified Oxford grading method was used for pelvic floor muscle assessment, and the evaluation criteria for pelvic floor muscle strength assessment (9) were as follows: grade I indicates that the vaginal muscles were slightly contracted during the test; grade II indicates that the vaginal muscles could maintain the contraction for 2 s and repeat it twice; grade III indicates that the vaginal muscles could maintain the contraction for 3 s and repeat it three times; grade IV indicates that the vaginal muscles could maintain the contraction for 4 s and repeat it four times; and grade V indicates that the vaginal muscles were significantly contracted that was able to sustain a contraction for 5 s or longer and repeated five or more times. These assessments were performed to collect comprehensive data on pelvic floor muscle pressure and strength in the study participants.

### Data analysis

**Figure 1:**
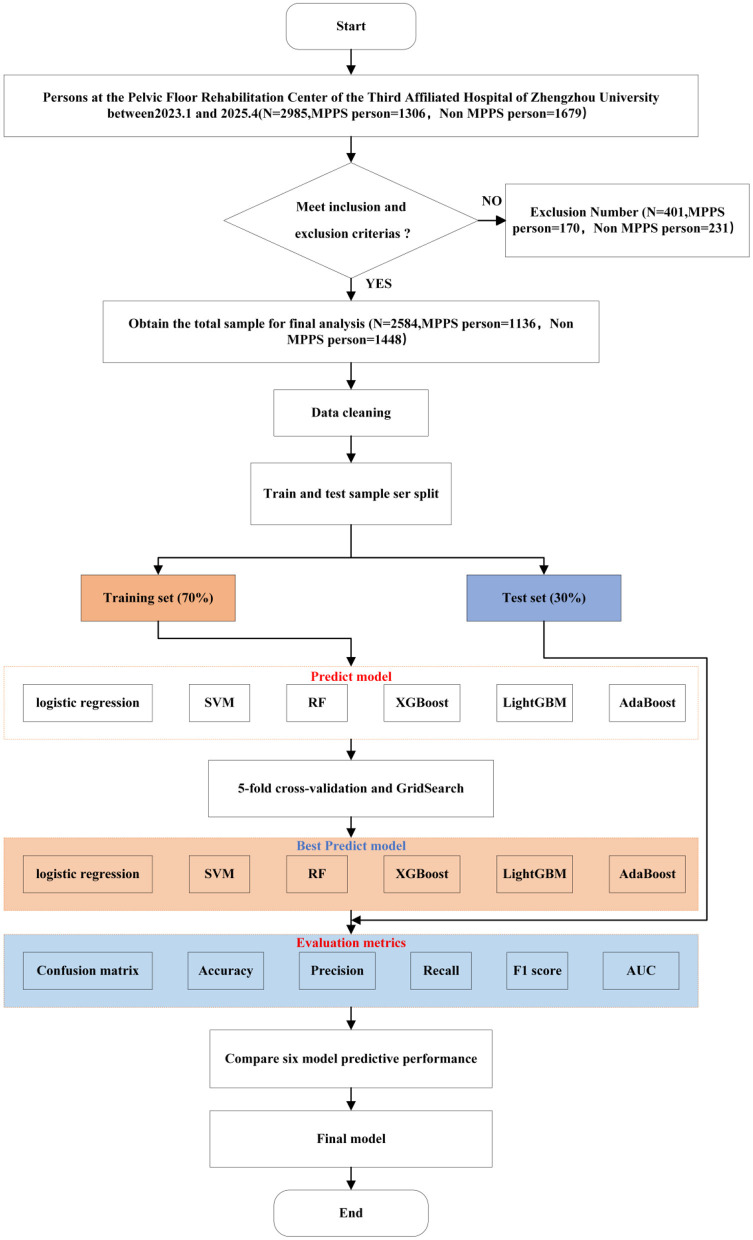
Process of modeling and analysis method.

Statistical analysis was performed using SPSS 26.0 and Python 3.9, Modeling and Analysis Flowchart 1 (Figure 1). A total of 23 clinical variables were included in this study (Table 1). Defective data were excluded from this study and after data cleaning, 2,584 individuals were included to form the final sample for analysis. The total sample was divided into a training data set and a test data set in a ratio of 7:3. Logistic, SVM, RF, XGBoost, LightGBM, and AdaBoost prediction models were built based on the training dataset, and a five-fold cross-validation was used to enhance the generalization performance of the model and avoid overfitting based on the test dataset. In this study, a five-fold cross-validation was carried out, and the training data were randomly divided into five parts, of which four were used for the model training, and one part was used for the model final performance evaluation. The above steps were repeated five times, and each iteration was randomly divided and selected. When all the iterations are completed, the mean and variance of the performance metrics of the five training sessions are calculated as the evaluation of the model performance. The performance of the six models is evaluated by confusion matrix, accuracy, recall, F1 score, and ROC curve based on the test dataset.

**Table 1:** Clinical characterization of MPPS and non-MPPS groups.

**Feature**	**Total number of people (*n* = 2,584 cases)**	**The non-MPPS group (*n* = 1,448 cases, 56%)**	**The MPPS group (*n* = 1,136 cases, 44%)**	**Efficiency value**	***P-*value**
Age (years)	36 (32, 44)	36 (32, 46)	36 (31, 43)	−4.21	< 0.001
Height (cm)	162 (158, 165)	162 (158, 165)	161 (158, 165)	−0.08	0.939
Weight (kg)	60 (54, 65)	60 (54, 65)	60 (53, 65)	−2.16	0.031
BMI (kg/m2)	22.59 (20.61, 25.00)	22.59 (20.76, 25.10)	22.59 (20.44, 24.83)	−2.21	0.027
Number of pregnancies (times)	3 (2, 4)	3 (2, 4)	3 (1, 4)	−2.34	0.019
Number of production (times)	1 (1, 2)	2 (1, 2)	1 (1, 2)	−5.09	< 0.001
**History of perineal laceration**					
No	2,530 (97.9%)	1,406 (97.1%)	1,124 (98.9%)	10.58	0.001
Yes	54 (2.1%)	42 (2.9%)	12 (1.1%)		
**History of hysterectomy**					
No	2,537 (98.1%)	1,409 (97.3%)	1,128 (99.3%)	14.1	< 0.001
Yes	47 (1.8%)	39 (2.7%)	8 (0.7%)		
Mean value of pre-resting phase (mmHg)	3.26 (2.65, 4.03)	3.18 (2.54, 4.03)	3.30 (2.94, 4.03)	−5.4	< 0.001
Coefficient of variation of pre-resting phase	0.13 (0.09, 0.19)	0.13 (0.09, 0.20)	0.13 (0.09, 0.19)	−1.81	0.07
Rapid systolic phase maximum (mmHg)	12.14 (8.55, 17.24)	12.04 (8.36, 17.38)	12.31 (8.78, 17.17)	−0.96	0.337
Relaxation time of rapid contraction phase (s)	1.59 (0.28, 8.02)	1.84 (0.28, 8.06)	1.41 (0.30, 8.02)	−0.97	0.334
Mean value of tense systolic phase (mmHg)	8.58 (6.54, 11.73)	9.14 (6.98, 11.60)	7.53 (6.22, 11.91)	−6.24	< 0.001
Coefficient of variation of tension-contraction phase	0.21 (0.15, 0.29)	0.21 (0.14, 0.28)	0.22 (0.15, 0.30)	−2.69	0.007
Relaxation time (s) during the tense contraction phase	9.00 (4.88, 9.00)	9.00 (5.46, 9.00)	9.00 (4.55, 9.00)	−1.5	0.134
Mean of endurance contraction phase (mmHg)	8.34 (6.73, 10.41)	8.98 (7.08, 11.24)	8.10 (6.42, 8.88)	−8.76	< 0.001
Coefficient of variation of endurance contraction phase	0.10 (0.07, 0.17)	0.10 (0.07, 0.16)	0.12 (0.07, 0.19)	−4.74	< 0.001
Post-resting phase mean (mmHg)	6.51 (5.13, 8.03)	6.57 (5.16, 8.06)	6.43 (5.07, 8.01)	−0.85	0.396
Post-resting stage coefficient of variation	0.04 (0.03, 0.06)	0.04 (0.03, 0.05)	0.05 (0.03, 0.06)	−7.05	< 0.001
Deep class I muscle strength	2 (1, 3)	2 (1, 3)	2 (1, 3)	−2.21	0.027
Deep class II muscle strength	3 (2, 3)	3 (2, 3)	3 (2, 3)	−1.73	0.084
Superficial class I muscle strength	2 (1, 3)	2 (1, 3)	2 (1, 3)	−2.02	0.044
Superficial class II muscle strength	3 (2, 3)	3 (2, 3)	3 (2, 4)	−2.66	0.008

Detailed descriptions of the evaluation procedures for both the Modified Oxford scale and Myotrac measurements, including rater information, training, and blinding.

Specifically:

Modified Oxford Scale: two experienced physiotherapists independently assessed pelvic floor muscle strength after standardized training. Inter- and intra-rater reliability were evaluated in a subset of 30 participants, yielding intra-class correlation coefficients (ICCs) of 0.87 and 0.83, respectively, indicating good reliability.

Myotrac Measurement Protocol: measurements were conducted in the lithotomy position, with three repeated contractions per subject. The average of the three readings was used for analysis. Raw signals were filtered (band-pass 20-500 Hz), and measurements were recorded at rest and during maximal voluntary contraction.

Blinding: all assessors were blinded to participant diagnostic labels and study group allocation to minimize bias.

## Results

**Figure 2:**
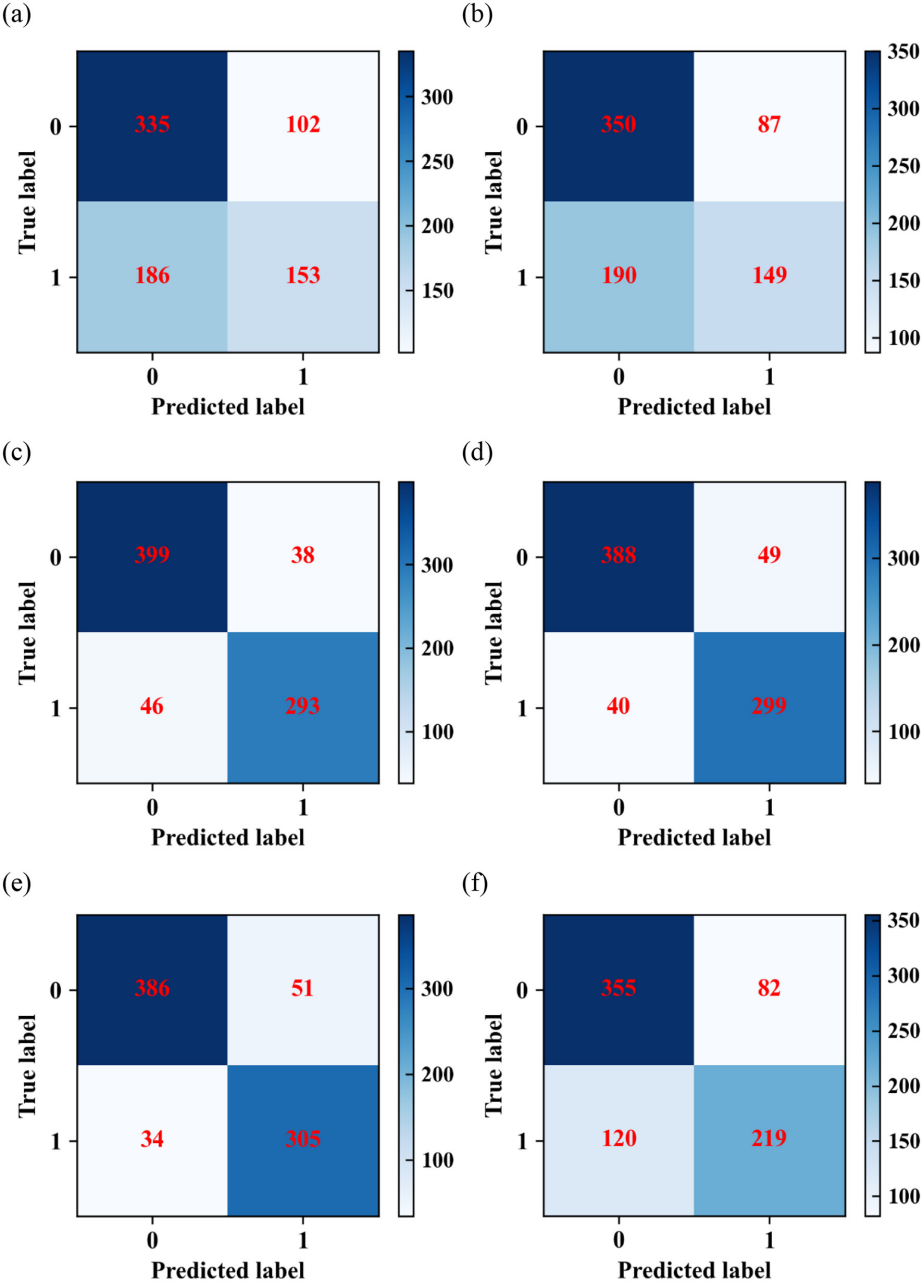
Confusion matrixes of **(a)** logistic regression; **(b)** SVM; **(c)** RF; **(d)** XGBoost; **(e)** LightGBM; and **(f)** AdaBoost algorithms.

(1) The confusion matrix for each model on the test dataset is given in Figure 2. In the data labeled 0, the logistic model predicts 335 cases correctly and 102 cases incorrectly; the SVM model predicts 350 cases correctly and 87 cases incorrectly; the RF model predicts 399 cases correctly and 38 cases incorrectly; the XGBoost model predicts 388 cases correctly and 49 cases incorrectly; the LightGBM model predicts 386 cases correctly and 51 cases incorrectly; AdaBoost model predicted 355 cases correctly and 82 cases incorrectly. In the data labeled 1, the logistic regression model predicted 153 cases correctly and 186 cases incorrectly; the SVM model predicted 149 cases correctly and 190 cases incorrectly; the RF model predicted 293 cases correctly and 46 cases incorrectly; the XGBoost model predicted 299 cases correctly and 40 cases incorrectly; the LightGBM model predicted 305 cases correctly and 34 cases incorrectly. The AdaBoost model predicted 219 cases correctly and 120 cases incorrectly.

(2) The performance of the six prediction models is analyzed in terms of accuracy, precision, recall, and F1 score. It is worth noting that this work solves a multi-class classification problem where each class has its precision, recall and F1 score, but only the overall accuracy is available.

**Figure 3:**
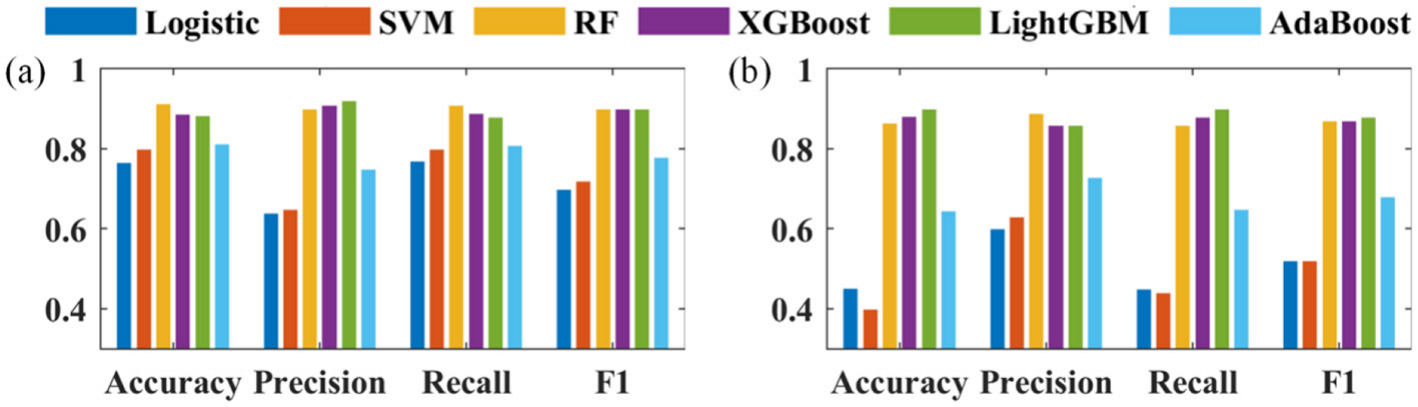
Evaluation indicators of **(a)** negative; **(b)** positive.

The performance of six machine learning models, logistic, SVM, RF, XGBoost, LightGBM, and AdaBoost, are evaluated based on the validation set using accuracy, precision, recall, and F1 scores, and the results are shown in Figure 3. The combined performance of RF, XGBoost, and LightGBM3 models are the best. Logistic regression, SVM, RF, XGBoost, LightGBM, AdaBoost 6 kinds of machine learning models performance of accuracy are respectively 0.77, 0.8, 0.91, 0.89, 0.88, 0.81, precisions are respectively 0.64, 0.65, 0.9, 0.91, 0.92, 0.75, recallings are respectively 0.77, 0.8, 0.91, 0.89, 0.88, 0.81, and F1 scores are respectively 0.7, 0.72, 0.9, 0.9, 0.9, 0.9, 0.78.

**Figure 4:**
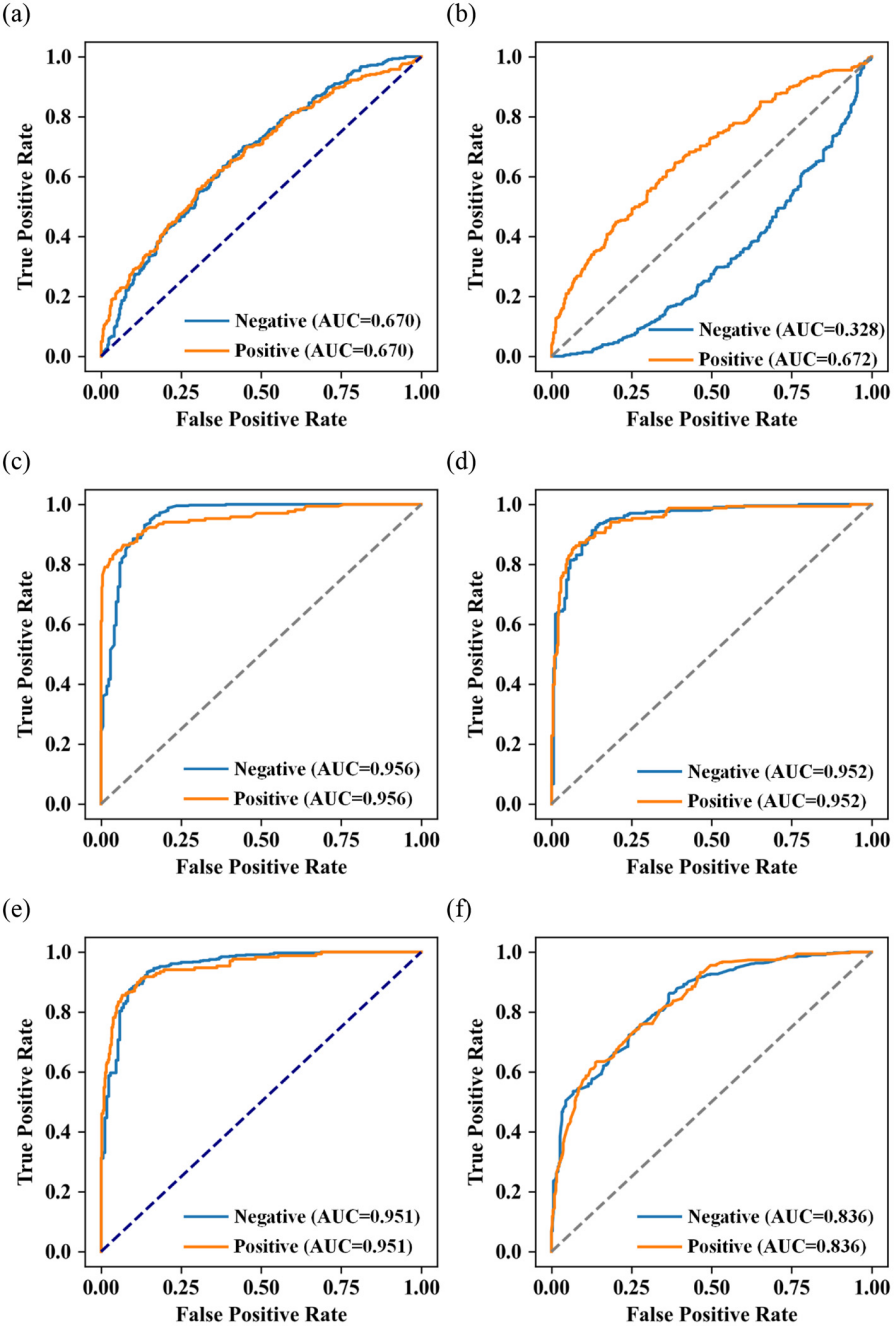
Receiver operating characteristic (ROC) of **(a)** logistic regression; **(b)** SVM; **(c)** RF; **(d)** XGBoost; **(e)** LightGBM; and **(f)** AdaBoost algorithms.

(3) The ROC curves of the five machine learning models are shown in Figure 4. Among all the models, RF performs the best (AUC = 0.956), XGBoost (AUC = 0.951) and LightGBM (AUC = 0.952) models perform slightly second, logistic regression (AUC = 0.670), SVM (AUC = 0.672), AdaBoost algorithms (AUC = 0.836) perform moderately well.

## Discussion

The most significant contribution of this work is to construct prediction models for various MPPS and compare their prediction performance, thereby identifying the model with better prediction performance. This study is grounded in the clinical variables of the patients enrolled in the research, in conjunction with assessments of pelvic floor pressure and data from the modified Oxford muscle strength evaluation. Then, we adapted SVM, RF, XGBoost, Light GBM and AdaBoost to establish the prediction models for MPPS. Through analyzing the prediction performance of each model based on confusion matrix, precision, recall, F1 score, overall accuracy, and ROC curve, we aim to look for the model that performs better in predicting the performance of MPPS. The results show that RF, XGBoost and LightGBM can be used to assist in the early diagnosis of MPPS. RF is the best performence for predicting MPPS among the six machine learning models, and the XGBoost and LightGBM models were slightly inferior.

MPPS is a kind of chronic pelvic pain, and the treatments of MPPS, both at home and abroad, are mostly based on physical means, supplemented by pain medications and psychological interventions, in order to maximize the preservation of the patient's physiological function and motor ability, and to improve the patient's quality of life (10). The mechanism of myofascial pain and myofascial trigger point formation in patients with MPPS is still unclear, and in recent years, with the increasing research related to MPPS, it has been found that central sensitization exists in most of the patients and leads to the problems of prolonged pain, delayed treatment, and unnecessary surgical interventions (11, 12). MPPS, if not effectively diagnosed and treated in the early stage, can further develop into urological, gynecological, and colorectal diseases or other musculoskeletal disorders. rectal diseases or other musculoskeletal disorders (13–15). However, the definition of MPPS is still unclear, laboratory tests and magnetic imaging are atypical, and the diagnosis needs to be consistent with the clinical symptoms and exclude other diseases that cause pelvic pain, which is difficult for clinicians, especially inexperienced young doctors (13, 14). Therefore, it is crucial to find a prediction model that can accurately differentiate whether or not one is suffering from MPPS. Correctly diagnosing MPPS at the early stage of the disease, avoiding misdiagnosis and omission, and developing a personalized treatment plan are conducive to enhancing patient compliance and improving treatment outcomes.

With the advancement of computer technology, machine learning methods have become well-known. Besides, the paper emphasizes that model interpretability and transparency are essential for clinician trust and adoption (16). Thanks to their flexible modeling capabilities, they exhibit advantages in depicting complex relationships between features and capturing non-linear patterns (16, 17). These advantages make them applicable to processing large-scale high-dimensional medical data (18), and they have now been widely introduced and applied in various fields of medicine. Machine algorithms have shown great application potential in etiological discovery, disease diagnosis, prediction of treatment effects, and construction of disease risk prediction models (19, 20), with impressive research outcomes. In this study, multiple machine learning algorithms were used to construct MPPS prediction models. This study was based on the clinical data of 2,584 patients who visited our hospital, divided into training and test sets in a ratio of 7:3. Six types of machine learning were used to analyze the clinical data in the training set and construct a prediction model about the key factors of MPPS occurrence, and a five-fold cross-validation validation set was used to enhance the generalization performance of the model and prevent model overfitting, and the performance of each model was evaluated using accuracy, precision, recall, F1 score and AUC. The results showed that the three models, RF, XGBoost, and LightGBM, performed well, and the AUC reached more than 0.95, which can strongly assist clinicians in the diagnosis of MPPS.

The logistic model in this study has the worst performance with AUC = 0.670, which is related to the fact that the logistic model cannot handle a large number of multi-class features or variables well, and is prone to be underfitted and inaccurate, despite its simplicity, ease of comprehension and implementation, it tends to perform poorly when there is a large feature space in a complex clinical problem (21). Decision tree models are easy to understand and have good interpretability, RF, XGBoost, LightGBM and AdaBoost models are meta classifiers constructed based on small decision trees (22–24). The performance of the five machine learning models constructed in this study is greatly improved compared to the logistic model, in which the first three have substantially improved performance, with AUCs greater than 0.95 and high confidence in the prediction probability, and AdaBoost is the next best, with AUC = 0.836. This may be due to the fact that AdaBoost, as an early Boosting algorithm, is noisy and outliers Extremely sensitive to noise and outliers, if there is noise or outliers in the data, its weight will be infinitely enlarged, and ultimately the generalization ability on the test set decreases dramatically; secondly, AdaBoost uses shallow decision trees with limited fitting ability, which makes it difficult to capture complex feature interactions. RF reduces the variance and improves the stability of the model by integrating multiple decision trees with randomly sampled features and samples (22), and the present study the RF model in this study performed best among the six models, with better accuracy, recall, and F1 score than other machine learning models. The article by Hang Yu et al. established an RF prediction model by analyzing clinical data from 1,245 cases, and compared it with a logistic model. The results suggested that the accuracy, sensitivity, specificity, and precision of the RF model were 87.11%, 90.66%, 90.91%, and 83.51%, respectively, and the AUC of the ROC curve was 0.942, which were better than that of the logistic model, and the RF model showed excellent predictive performance in the initial screening assessment of MPPS diseases (7). The RF model has a stable performance and a high credibility of the predictive probability, which is expected to be used in the clinic to provide more reliable decision support for clinicians. It is expected to be used in clinical settings to provide clinicians with more reliable decision support. The results of the study showed that the Random Forest model demonstrated superior clinical predictive performance in identifying risk factors in MPPS patients, which provides important support and guidance for enhancing the early identification and personalized treatment of MPPS. The RF model demonstrated good predictive performance in this single-center retrospective study. However, before it can be applied in clinical settings, external validation and prospective studies are necessary to confirm its generalizability and clinical utility. In the early stages of MPPS, treatment is inexpensive and requires only rehabilitation without clinical intervention. Patients at this stage often exhibit subtle symptoms, posing a significant diagnostic challenge to healthcare professionals. With the predictive model proposed in this study, timely diagnosis of MPPS in its early stages can be achieved, stopping the progression of the disease and eliminating the need for clinical intervention. Such a model would help reduce the economic burden, improve the quality of life of patients, and is essential for improving the efficiency and quality of healthcare delivery. Patient outcomes and quality of life can be significantly improved through accurate prediction and timely intervention.

Despite the excellent performance of the model, the following limitations should be noted: the small sample size in rural areas and single-center study may lead to selection bias and affect the extrapolation applicability of the model. Future improvements can be made by continuously updating the database to enhance stability, expanding the sample size and multicenter data collection, and increasing the diversity of predictor variables, as well as combining external validation to provide more reliable clinical decision support through continuous optimization.

## Conclusion

The RF model is the best performing model for predicting MPPS among the six machine learning models, with the XGBoost and LightGBM models slightly following, and the AUC of all three is greater than 0.95, which can be used to assist in the diagnosis of MPPS and to help clinicians screen for MPPS early, so as to provide individualized and differentiated treatments for patients.

## Data Availability

The raw data supporting the conclusions of this article will be made available by the authors, without undue reservation.

## References

[1] Bonder JH Chi M Rispoli L. Myofascial pelvic pain and related disorders. Phys Med Rehabil Clin N Am. (2017) 28:501–15. doi: 10.1016/j.pmr.2017.03.005 28676361

[2] Ross V Detterman C Hallisey A. Myofascial pelvic pain: an overlooked and treatable cause of chronic pelvic pain. Midwifery Womens Health. (2021) 66:148–60. doi: 10.1111/jmwh.13224 33788379

[3] Cao QW Peng BG Wang L Huang YQ Jia DL Jiang H . Expert consensus on the diagnosis and treatment of myofascial pain syndrome. World J Clin Cases. (2021) 9:2077–89. doi: 10.12998/wjcc.v9.i9.2077 33850927 PMC8017503

[4] Afzal U Saeed Q Anwar MN Pervaiz S Shahid M Javed R . Comparison of health parameters in postpartum diastasis recti: a randomized control trial of SEMG biofeedback-assisted core strengthening exercises with kinesiotaping vs. non-assisted exercises. Healthcare. (2024) 12:1567. doi: 10.3390/healthcare12161567 39201126 PMC11354019

[5] Chen ZH Lin L Wu CF Li CF Xu RH Sun Y. Artificial intelligence for assisting cancer diagnosis and treatment in the era of precision medicine. Cancer Commun. (2021) 41:1100–15. doi: 10.1002/cac2.12215 34613667 PMC8626610

[6] Yang X Wang Y Byrne R Schneider G Yang S. Concepts of artificial intelligence for computer-assisted drug discovery. Chem Rev. (2019) 119:10520–94. doi: 10.1021/acs.chemrev.8b00728 31294972

[7] Yu H Zhao H Liu D Dong Y Nai M Song Y . Prediction of myofascial pelvic pain syndrome based on random forest model. Heliyon. (2024) 10:e31928. doi: 10.1016/j.heliyon.2024.e31928 38868063 PMC11167342

[8] Akhmedzhanova LT Leontyeva MS Mandra EV Barinov AN. Diagnosis and treatment of chronic pelvic pain syndrome, neurology, neuropsychiatry. Psychosomatics. (2022) 14:54–61. doi: 10.14412/2074-2711-2022-4-54-61

[9] Klotz SGR Schön M Ketels G Löwe B Brünahl CA. Physiotherapy management of patients with chronic pelvic pain (CPP). A systematic review. Physiother Theory Pract. (2019) 35:516–32. doi: 10.1080/09593985.2018.1455251 29589778

[10] Aredo JV Heyrana KJ Karp BI Shah JP Stratton P. Relating chronic pelvic pain and endometriosis to signs of sensitization and myofascial pain and dysfunction. Semin Reprod Med. (2017) 35:88–97. doi: 10.1055/s-0036-1597123 28049214 PMC5585080

[11] Lamvu G Carrillo J Witzeman K Alappattu M. Musculoskeletal considerations in female patients with chronic pelvic pain. Semin Reprod Med. (2018) 36:107–15. doi: 10.1055/s-0038-1676085 30566976

[12] Jarrell JF Vilos GA Allaire C Burgess S Fortin C Gerwin R . No. 164—Consensus guidelines for the management of chronic pelvic pain. J Obstet Gynaecol CA. (2018) 40:e747–87. doi: 10.1016/j.jogc.2018.08.015 30473127

[13] He HJ Chen J Hou Z Duan H Zhang P Lu GJ . Expert consensus on the clinical management of chronic pelvic pain in women (2021 edition). Beijing Med. (2021) 7:650–9. doi: 10.3760/cma.j.cn112141-20240320-00171

[14] Mardon AK Leake HB Chalmers KJ. A review of chronic pelvic pain in women. J Am Med Assoc. (2021) 326:2206. doi: 10.1001/jama.2021.17977 34874427

[15] Cummings M Baldry P. Regional myofascial pain: diagnosis and management. Best Pract Res Clin Rheumatol. (2007) 21:367–87. doi: 10.1016/j.berh.2006.12.006 17512488

[16] Abbas Q Jeong W Lee SW. Explainable AI in clinical decision support systems: a meta-analysis of methods, applications, and usability challenges. Healthcare. (2025) 13:2154. doi: 10.3390/healthcare13172154 40941506 PMC12427955

[17] Ruffle JK Tinkler L Emmett C Ford AC Nachev P Aziz Q . Constipation predominant irritable bowel syndrome and functional constipation are not discrete disorders: a machine learning Approach. Am J Gastroenterol. (2021) 116:142–51. doi: 10.14309/ajg.0000000000000816 32868630

[18] Elgendi M Allaire C Williams C Bedaiwy MA Yong PJ. Machine learning revealed new correlates of chronic pelvic pain in women. Front Digit Health. (2020) 2:600604. doi: 10.3389/fdgth.2020.600604 34713065 PMC8521902

[19] Choi JY Lee JH Choi Y Hyon Y Kim YH. Prediction of disorders with significant coronary lesions using machine learning in patients admitted with chest symptoms. PLoS ONE. (2022) 17:e0274416. doi: 10.1371/journal.pone.0274416 36215242 PMC9550076

[20] Celeste C Ming D Broce J Ojo DP Drobina E Louis-Jacques AF . Ethnic disparity in diagnosing asymptomatic bacterial vaginosis using machine learning. NPJ Digit Med. (2023) 6:211. doi: 10.1038/s41746-023-00953-1 37978250 PMC10656445

[21] Handelman GS Kok HK Chandra RV Razavi AH Lee MJ Asadi H. eDoctor: machine learning and the future of medicine. J Intern Med. (2018) 284:603–19. doi: 10.1111/joim.12822 30102808

[22] Roy SS Dey S Chatterjee S. Autocorrelation-aided random forest classifier-based bearing fault detection framework. IEEE Sens J. (2020) 20:10792–800. doi: 10.1109/JSEN.2020.2995109

[23] Javed RA Jalil Z Moqurrab SA Abbas S Liu X. Ensemble AdaBoost classifier for accurate and fast detection of botnet attacks in connected vehicles. Trans Emerg Telecommun Technol. (2022) 3:e4088. doi: 10.1002/ett.4088

[24] Yu D Liu Z Su C Han Y Duan X Zhang R . Copy number variation in plasma as a tool for lung cancer prediction using the Extreme Gradient Boosting (XGBoost) classifier. Thoracic cancer. (2020) 11:95–102. doi: 10.1111/1759-7714.13204 PMC6938748 31694073

